# Field-Scale Suppression of Cyanobacterial Blooms by Cationic Polymer-Modified Local Soil: Selective Inactivation Mechanism and Ecological Responses

**DOI:** 10.3390/microorganisms14092111

**Published:** 2026-09-21

**Authors:** Ningyan Peng, Yuanyuan Fang, Ping Yang, Jin Liu, Yunlu Jia, Zhengwei Dai, Xiaojin Zhang, Renjie Zhao, Shouchun Li, Guofei Dai

**Affiliations:** 1 Key Laboratory of Watershed Soil and Water Conservation, Technology Innovation Center for Ecological Water Engineering in Poyang Lake, Jiangxi Academy of Water Science and Engineering, Nanchang 330029, China; 2 Institute of Hydrobiology, Chinese Academy of Sciences, Wuhan 430072, China; 3 Faculty of Materials Science and Chemistry, China University of Geosciences, Wuhan 430074, China; 4 College of Life Science, Jiangxi Normal University, Nanchang 330022, China

**Keywords:** harmful cyanobacterial bloom, *Microcystis*, cationic polyquaternium, modified local soil, electrostatic interaction, field case study

## Abstract

Harmful cyanobacterial blooms require control strategies that suppress bloom-forming taxa while limiting effects on non-target primary producers and toxin-related risks. We developed a cationic polyquaternium (P126)-engineered local red-soil composite and evaluated its electrokinetic properties, taxon-dependent photophysiological effects, activity against colonial *Microcystis*, robustness to water chemistry, short-term *Danio rerio* responses, and field-scale performance. P126 modification reversed soil surface charge to the range of +20 to +33 mV across a pH range of 6.0–10.0, generating a strong electrostatic contrast with negatively charged cyanobacteria. At 10 mg L^−1^, the composite suppressed maximum photosystem II quantum yield (F_v_/F_m_) in *Microcystis aeruginosa* and *Dolichospermum* sp., with no statistically detectable effects on the four tested chlorophyte and diatom strains. For *Microcystis* colonies ≥ 500 μm, 20 mg L^−1^ of composite achieved >95% MTT-based inhibition within 24 h, versus < 15% for CuSO_4_. Suppression persisted across the tested pH and nutrient ranges but was attenuated by high humate concentrations. The maximum unfractionated-sample ELISA microcystin-equivalent signal was approximately 25% lower than with CuSO_4_; however, dissolved and particulate fractions were not resolved. No statistically detectable treatment-related differences in zebrafish hatching or larval length were observed up to 10.5 mg L^−1^. In a physically isolated sub-lake, composite application preceded a decline of more than three orders of magnitude in algal/cyanobacterial density and an increase in Secchi depth from approximately 0.25 to 1.2 m; *Vallisneria natans* was planted subsequently. These results provide laboratory evidence and field case-study observations consistent with electrostatically favored, selective bloom suppression, while residual-polymer fate, sediment effects, chronic toxicity, and multi-trophic responses require further evaluation.

## 1. Introduction

Harmful cyanobacterial blooms are an increasing threat to freshwater ecosystems under persistent eutrophication and climate-related hydrological change [1,2,3]. Dense cyanobacterial biomass alters light and oxygen regimes and disrupts food-web processes, while microcystins and other cyanotoxins can compromise drinking water and recreational uses; their persistence and treatment further complicate bloom management [4,5,6,7]. Effective interventions must therefore reduce bloom biomass while limiting secondary ecological and toxicological effects.

Copper sulfate (CuSO_4_) remains a widely used chemical treatment for bloom control [8]. However, copper is not taxonomically selective and can affect non-target primary producers [9,10]; following lake application, dissolved copper can partition to particulate material and sediment [8]. Rapid cellular damage may also increase the concentration of microcystins in the water phase [11]. These limitations have motivated interest in physical and physicochemical approaches that can remove or suppress cyanobacteria with lower non-target exposure [12,13].

Clay- and soil-based flocculation can transfer cyanobacterial biomass from the water column to sediments [14,15,16,17]. Nevertheless, unmodified mineral particles mainly provide ballast, and viable cells deposited at the sediment–water interface may contribute to later bloom recruitment after resuspension [16,17]. Cationic modifiers can strengthen aggregation and contact with negatively charged cyanobacterial surfaces [18,19,20,21], but freely dissolved quaternary ammonium compounds and related polycations have raised concerns regarding persistence, sorption, transformation, and chronic effects in aquatic environments [22,23,24,25,26].

A risk-aware material design should therefore combine targeted efficacy with reduced exposure of freely dissolved cationic agents. Associating a cationic polymer with an abundant mineral carrier may localize the active functionality and promote sedimentation, but surface association does not demonstrate complete immobilization or environmental inertness. Polymer-specific measurements, sediment fate, and multi-trophic effects remain necessary components of ecological evaluation.

Here, we engineered locally sourced red soil with the cationic polyquaternium polymer P126. We hypothesized that the resulting positively charged composite would preferentially associate with the strongly negative extracellular matrix of bloom-forming cyanobacteria. The objectives were to (1) characterize electrokinetic differences among the composite and representative phytoplankton; (2) evaluate taxon-dependent effects on photosynthetic performance; (3) compare composite and CuSO_4_ performance against *Microcystis* colonies and their associated unfractionated-sample ELISA microcystin-equivalent signals; (4) assess short-term developmental endpoints in *Danio rerio* early life stages; (5) test the influence of relevant water-chemistry conditions; and (6) document field-scale ecological responses while explicitly defining the limits of causal and safety interpretation.

## 2. Materials and Methods

### 2.1. Materials and Reagents

Red soil was obtained from a terrestrial site adjacent to Poyang Lake, Jiangxi Province, China. The soil was air-dried, ground, and passed through a 200-mesh sieve. Its physicochemical characteristics and Fe, Cu, Mg, and total P contents are provided in Supplementary Tables S1 and S2. The cationic polyquaternium polymer P126 (approximate molecular mass, 30 kDa; CAS Registry No. 31075-24-8) was obtained from Copolymer Chemical Corporation (Suzhou, China). Copper(II) sulfate pentahydrate (CuSO_4_·5H_2_O, analytical grade), potassium nitrate, potassium dihydrogen phosphate, and sodium humate used as a humic-substance surrogate were obtained from commercial suppliers.

### 2.2. Preparation of the P126-Engineered Soil Composite

An aqueous P126 stock solution was added manually and dropwise to a mechanically stirred suspension of red soil (10 g L^−1^ in deionized water) while stirring at 200 rpm to obtain a P126-to-soil input mass ratio of 1:4. The 2 h mixing interval began after addition was complete. Because a calibrated dosing pump was not used, the volumetric addition rate and exact addition duration were not recorded. The suspension was then allowed to settle for 1 h. The supernatant was decanted, and the recovered solid was resuspended in deionized water, allowed to settle, and decanted twice to reduce readily soluble P126. The washed solid was dried at 50 °C and gently ground. Raw red soil subjected to the same preparation procedure without P126 was prepared as the unmodified material comparator for zeta-potential characterization. Composite concentrations reported below refer to the nominal mass concentration of the complete P126-engineered solid, not the mass concentration of P126 alone. Replication numbers refer to independent biological assays; material preparation followed the procedure described here.

### 2.3. Phytoplankton Strains and Cultivation

Six phytoplankton strains were obtained from the Freshwater Algae Culture Collection of the Institute of Hydrobiology (FACHB), China. Cyanobacteria comprised *Microcystis aeruginosa* (FACHB-1322) and *Dolichospermum* sp. (FACHB-1091). Non-target taxa comprised the chlorophytes *Chlorella* sp. (FACHB-8) and *Scenedesmus quadricauda* (FACHB-44) and the diatoms *Nitzschia palea* (FACHB-2263) and *Melosira varians* (FACHB-2599). Cyanobacteria and chlorophytes were maintained in BG-11 medium, and diatoms were maintained in CSI medium, at 25 ± 1 °C under 40 μmol photons m^−2^ s^−1^ and a 12:12 h light/dark cycle. Exponential-phase cultures were used in the experiments.

### 2.4. Analytical and Biological Assays

#### 2.4.1. Chlorophyll Fluorescence

Photophysiological performance was measured using the four-wavelength PHYTO-PAM Phytoplankton Analyzer (Heinz Walz GmbH, Effeltrich, Germany) using the 470 nm measuring-light channel. The pulse-modulated measuring light consisted of 12 μs LED pulses. Aliquots (3 mL) were dark-adapted for 20 min before minimum (F_0_) and maximum (F_m_) fluorescence were recorded. Maximum PSII quantum yield was calculated as F_v_/F_m_ = (F_m_ − F_0_)/F_m_. Cultures with an initial density of approximately 5 × 10^8^ cells L^−1^ were exposed to 0, 2.5, 5, or 10 mg L^−1^ composite and monitored over 96 h. In this study, inhibition or inactivation denotes a loss of measurable physiological activity rather than sedimentation alone.

#### 2.4.2. MTT-Based Metabolic Viability

The metabolic viability of *Microcystis* was evaluated using the MTT [3-(4,5-dimethylthiazol-2-yl)-2,5-diphenyltetrazolium bromide] assay. Suspensions with an initial density of approximately 5 × 10^8^ cells L^−1^ were separated into large colonies (diameter ≥500 μm) and single cells or small colonies (diameter ≤ 60 μm) and treated with 0, 5, 10, or 20 mg L^−1^ P126-engineered composite or CuSO_4_. MTT solution was added after treatment, samples were incubated at 35 °C for 1 h in darkness, formazan was dissolved in dimethyl sulfoxide, and absorbance was measured at 556 nm. Relative viability was normalized to the corresponding untreated control, and inhibition efficiency was expressed as 100 × (1 − treated/control viability). Measurements were obtained over 5 d. 

#### 2.4.3. Zeta-Potential Measurements

Surface electrokinetic properties were measured using a Zetasizer Nano ZS (Malvern Instruments, Malvern, UK). Raw soil, P126-engineered composite, phytoplankton cultures, and a natural bloom sample were dispersed in deionized water; no supporting electrolyte was deliberately added (nominal added concentration, 0 mM). The pH was adjusted using small aliquots of 0.1 M HCl or NaOH. Consequently, actual ionic strength was not standardized and included sample-derived ions and titrant additions. Measurements were conducted at 25.0 °C (manufacturer-specified temperature-control accuracy, ±0.1 °C at 25 °C) in triplicate. No run-specific measurement of an external zeta-potential transfer standard was retained; therefore, no run-specific standard value is reported.

#### 2.4.4. Water Quality, Phytoplankton Enumeration, and Microcystin Analysis

Total nitrogen (TN), total phosphorus (TP), ammonia nitrogen (NH_4_–N), and orthophosphate (PO_4_–P) were analyzed according to APHA Standard Methods for the Examination of Water and Wastewater, 23rd edition [27]. Water samples collected from the treated and reference basins at each monitoring date were examined by microscopic enumeration using a Sedgewick–Rafter counting chamber. The available field dataset supported longitudinal abundance estimates but did not provide a complete, consistently resolved percentage composition of chlorophytes and diatoms.

Microcystin-related responses were measured using a Microcystin Plate Kit (catalog No. 125011; FACHB, Institute of Hydrobiology, Chinese Academy of Sciences, Wuhan, China; manufacturer-reported detection limit, 0.1 μg L^−1^). A method-specific limit of quantification was not independently established. Samples were analyzed as unfractionated suspensions; paired filtrates, centrifuged supernatants, and pellets were not generated. Because an ELISA does not distinguish individual congeners and the experimental record did not support separation of dissolved and particulate fractions, results are reported as unfractionated-sample microcystin-equivalent signals rather than congener-resolved or strictly dissolved concentrations. For the comparison, large *Microcystis* colonies (≥500 μm; approximately 5 × 10^8^ cells L^−1^) were treated with 5 mg L^−1^ of composite or 5 mg L^−1^ of CuSO_4_ and monitored for 5 d.

### 2.5. Influence of Environmental Conditions

Batch assays were conducted in 100 mL suspensions of a diluted natural *Microcystis* bloom sample from Poyang Lake (initial density, approximately 7.5 × 10^7^ cells L^−1^) on an orbital shaker at 100 rpm. The following factors were varied individually: pH of 6.0–10.0; nitrogen added as KNO_3_ to 0, 1, 5, 25, or 50 mg L^−1^ of N; phosphorus added as KH_2_PO_4_ to 0, 0.1, 0.25, 1, or 10 mg L^−1^ of P; and sodium humate to 0, 1, 5, 20, or 100 mg L^−1^. The composite was applied at 5 mg L^−1^, and F_v_/F_m_ and chlorophyll-a were monitored over 5 d.

### 2.6. Danio Rerio Early-Life-Stage Assay

Short-term developmental endpoints were assessed using *Danio rerio* embryos in E3 medium at 26 ± 1 °C under a 14:10 h light/dark cycle. Fertilized embryos were allocated at the beginning of exposure, with 30 individuals per replicate and three replicates per treatment. The exact hours post-fertilization and corresponding developmental stage at exposure initiation were not retained in the archived study record; therefore, the exact exposure duration before the 72- and 96 h post-fertilization endpoints cannot be reconstructed. Hatching success was evaluated at composite concentrations of 0, 1.75, 3.5, 7.0, and 10.5 mg L^−1^ at 72 h post-fertilization. Larval body length was evaluated at 0, 3.5, 7.0, and 10.5 mg L^−1^ at 96 h post-fertilization. These endpoints were used to assess short-term responses and were not interpreted as a comprehensive chronic, benthic, or ecosystem-level safety test.

### 2.7. Field Implementation and Ecological Monitoring

The field study was conducted by the project team in a physically isolated hypertrophic sub-lake within the Poyang Lake system (area, 1.4 ha; mean depth, 1.4 m). The intervention formed part of the lake-management research project identified in the Funding section. In September 2023, the P126-engineered composite was distributed from boat-mounted applicators in four nominal applications of approximately 11.25 mg L^−1^ composite each (twice weekly for two weeks), giving a cumulative nominal dose equivalent to approximately 45 mg L^−1^ when referenced to basin water volume. This fractionated operational dose was not established as a chronic no-effect threshold. After pelagic turbidity was reduced, the submerged macrophyte *Vallisneria natans* was deliberately planted as a separate restoration measure. A hydrologically separated basin within the same lake system was monitored as a reference from March 2023 to March 2024. The field design comprised one treated and one reference basin and was therefore interpreted as a longitudinal operational case study rather than a replicated basin-level experiment.

### 2.8. Statistical Analysis

Laboratory data are presented as the mean ± standard deviation from three independent biological replicates unless otherwise stated. Treatment effects at individual sampling times were evaluated by one-way analysis of variance followed by Tukey’s HSD test, with *p <* 0.05 considered statistically significant. Formal normality and homogeneity-of-variance testing were not performed. Given the small group size (*n* = 3), inferential results are interpreted as exploratory; *p* > 0.05 is described only as no statistically detectable difference and not as evidence of equivalence. Analyses were performed in OriginPro 7.5. Because the field component included one treated and one reference basin, field time series were interpreted descriptively, no basin-level inferential test was applied, and repeated sampling dates were not treated as independent basin replicates.

## 3. Results

### 3.1. Electrokinetic Differentiation

**Figure 1:**
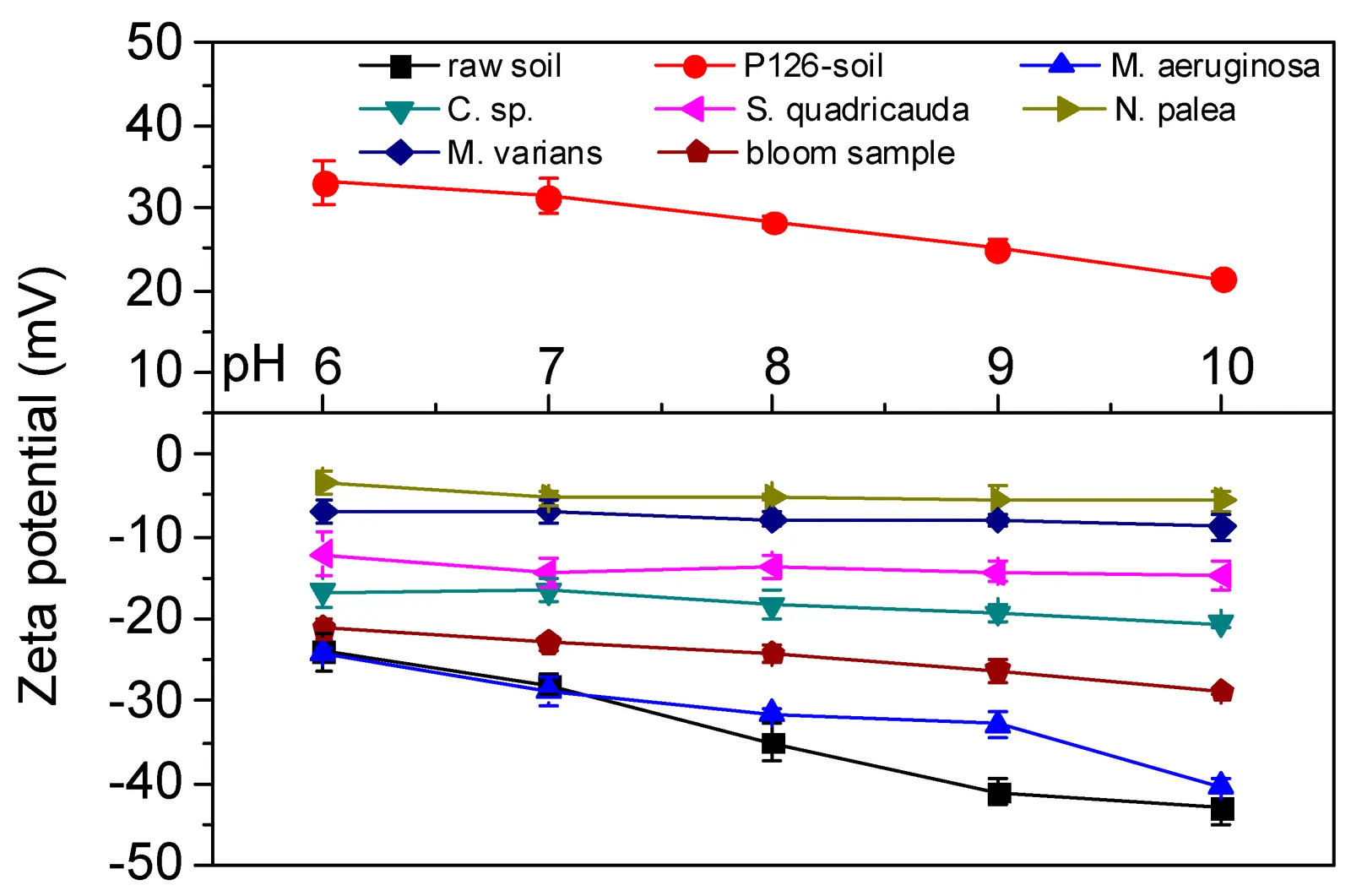
Zeta potentials of raw soil, the P126-engineered composite, representative phytoplankton strains, and a natural bloom sample across pH of 6.0–10.0. Data are shown as mean ± SD (*n* = 3).

Raw red soil became progressively more negative across pH 6.0–10.0, from approximately −24 mV at pH 6.0 to −44 mV at pH 10.0, whereas the P126-engineered composite showed charge reversal and decreased from approximately +33 mV at pH 6.0 to +20 mV at pH 10.0 (Figure 1). This shift was consistent with retained cationic surface functionality after composite preparation.

The tested phytoplankton displayed distinct electrokinetic profiles. *Microcystis aeruginosa* was the most negative biological sample, ranging from approximately −24 mV at pH 6.0 to −41 mV at pH 10.0, whereas the natural bloom sample ranged from approximately −21 to −30 mV. Chlorophytes showed intermediate values (approximately −12 to −21 mV), whereas the diatoms were weakly negative (approximately −3 to −9 mV). These differences provided an electrokinetic basis for stronger association between the positive composite and the tested cyanobacteria.

### 3.2. Taxon-Dependent Photophysiological Responses

**Figure 2:**
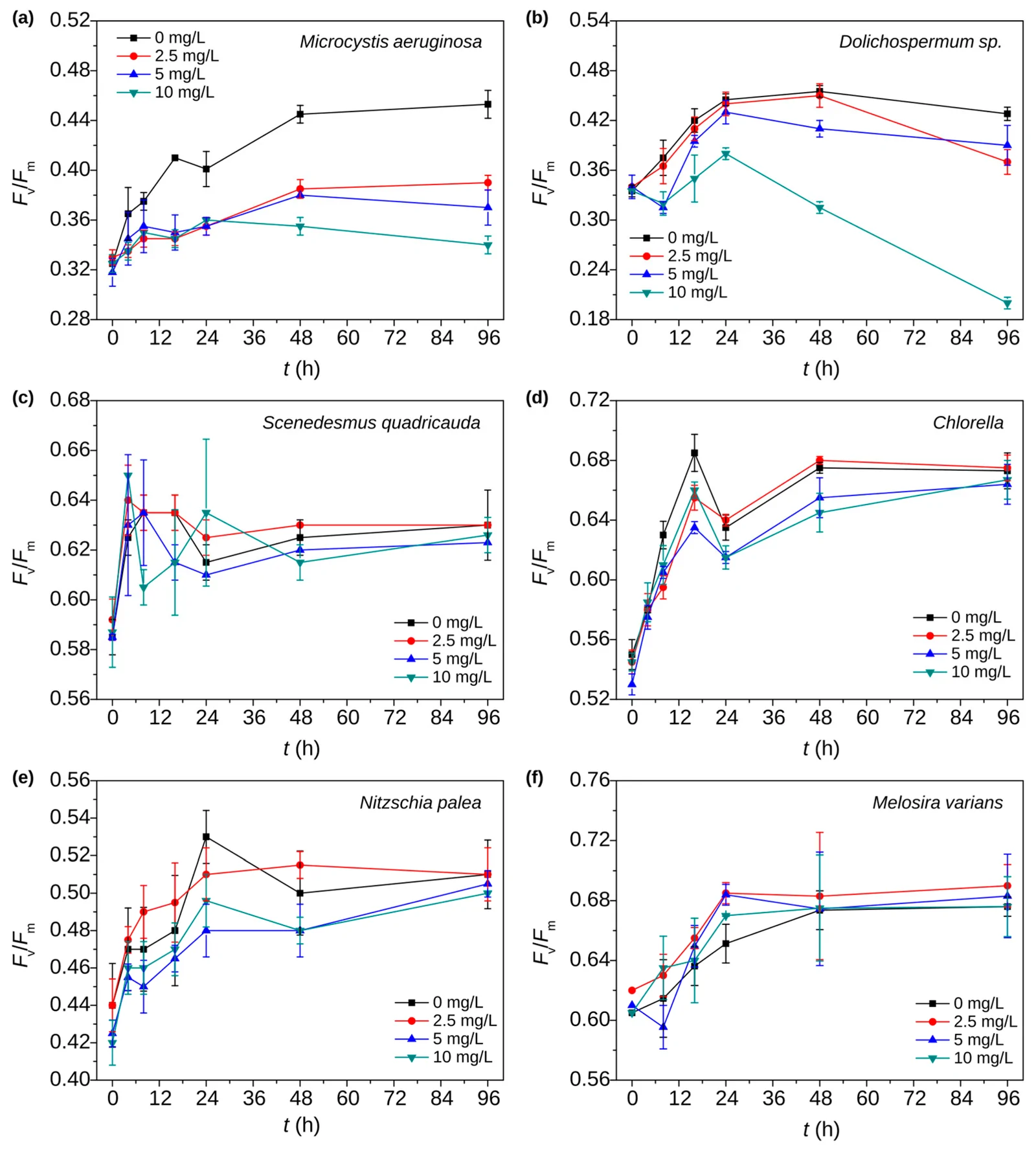
Maximum PSII quantum yield (F_v_/F_m_) of (**a**) *Microcystis aeruginosa*, (**b**) *Dolichospermum* sp., (**c**) *Scenedesmus quadricauda*, (**d**) *Chlorella* sp., (**e**) *Nitzschia palea*, and (**f**) *Melosira varians* exposed to 0, 2.5, 5, or 10 mg L^−1^ of P126-engineered composite over 96 h. Data are shown as mean ± SD (*n* = 3).

Composite exposure produced dose- and time-dependent changes in the two cyanobacteria (Figure 2a,b). At 10 mg L^−1^, F_v_/F_m_ remained below the corresponding controls at 96 h, with the strongest decline observed for *Dolichospermum* sp. *Microcystis aeruginosa* also showed sustained suppression relative to its untreated control.

In contrast, the F_v_/F_m_ trajectories of *Scenedesmus quadricauda*, *Chlorella* sp., *Nitzschia palea*, and *Melosira varians* showed no statistically detectable treatment effect at the corresponding sampling times under the tested concentrations (exploratory *p >* 0.05; Figure 2c–f). The result therefore supports selectivity among the six tested strains but should not be generalized to all non-target phytoplankton.

### 3.3. Performance Against Colonial Microcystis and ELISA Microcystin Signals

**Figure 3:**
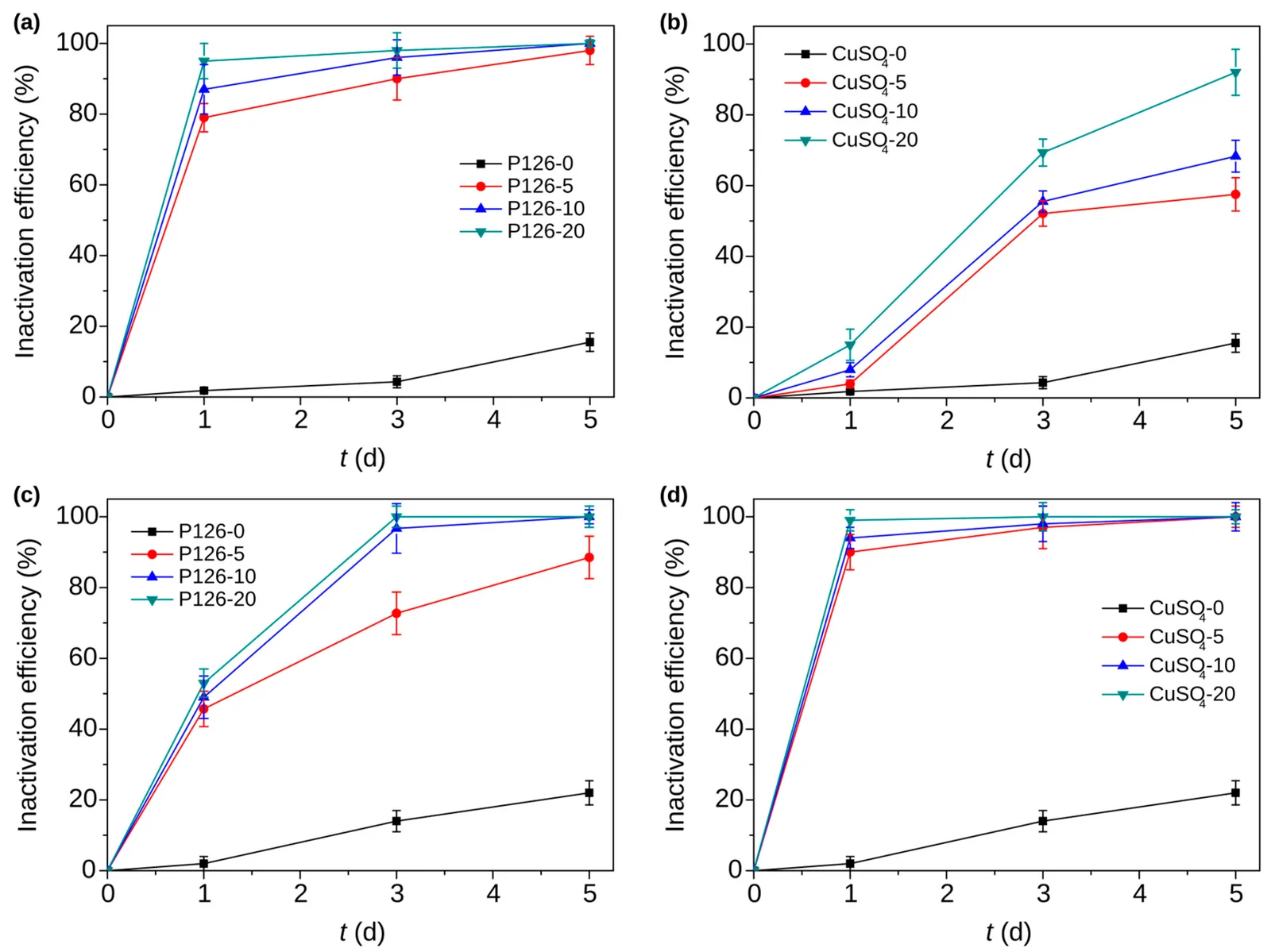
MTT-based inhibition of *Microcystis* by the P126-engineered composite and CuSO_4_. Panels (**a**,**b**) show large colonies (diameter ≥ 500 μm), and panels (**c**,**d**) show single cells/small colonies (diameter ≤ 60 μm) at nominal treatment concentrations of 0, 5, 10, or 20 mg L^−1^. Data are shown as mean ± SD (*n* = 3).

Large *Microcystis* colonies were less responsive to CuSO_4_ than to the composite (Figure 3a,b). At 20 mg L^−1^, the composite produced >95% MTT-based inhibition within 24 h, whereas CuSO_4_ at the same nominal mass concentration produced <15% inhibition. For single cells and small colonies (≤60 μm), both treatments produced high inhibition, with near-complete responses at the higher concentrations by days 3–5 (Figure 3c,d).

**Figure 4:**
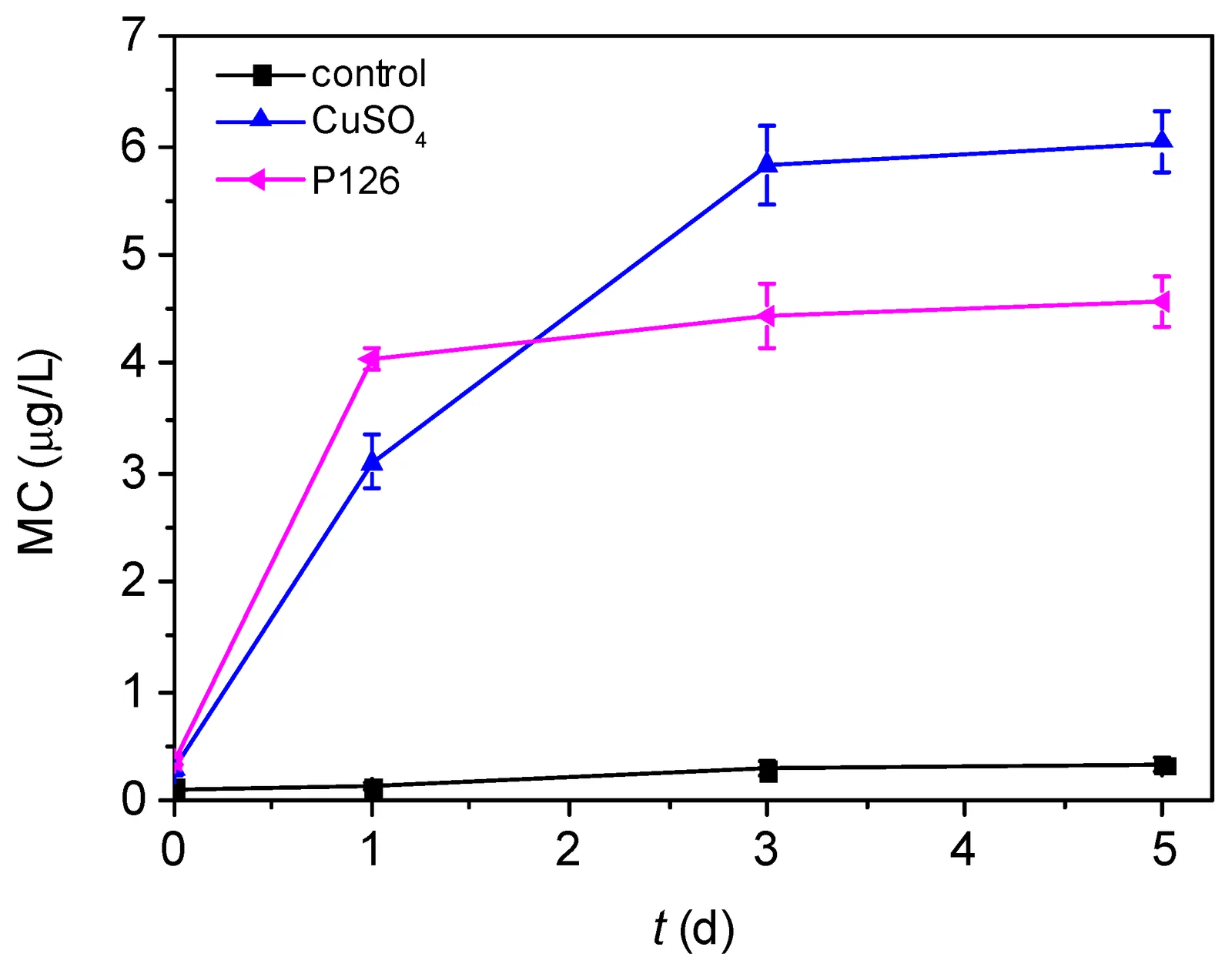
Unfractionated-sample ELISA microcystin-equivalent signals after treatment of large *Microcystis* colonies (≥500 μm; approximately 5 × 10^8^ cells L^−1^) with 5 mg L^−1^ of P126-engineered composite or 5 mg L^−1^ of CuSO_4_. Samples were analyzed without filtration or centrifugation; the assay did not resolve individual congeners or dissolved and particulate fractions. Data are shown as mean ± SD (*n* = 3).

The maximum unfractionated-sample ELISA microcystin-equivalent signal was approximately 4.6 μg L^−1^ in the composite treatment and 6.1 μg L^−1^ in the CuSO_4_ treatment (Figure 4), corresponding to an approximately 25% lower maximum signal for the composite under the tested conditions. Because samples were not fractionated, this comparison cannot distinguish intracellular, floc-associated, and freely dissolved microcystin, and it does not demonstrate reduced cell lysis or reduced dissolved-toxin release.

### 3.4. Effects of pH, Nutrients, and Humic Substances

**Figure 5:**
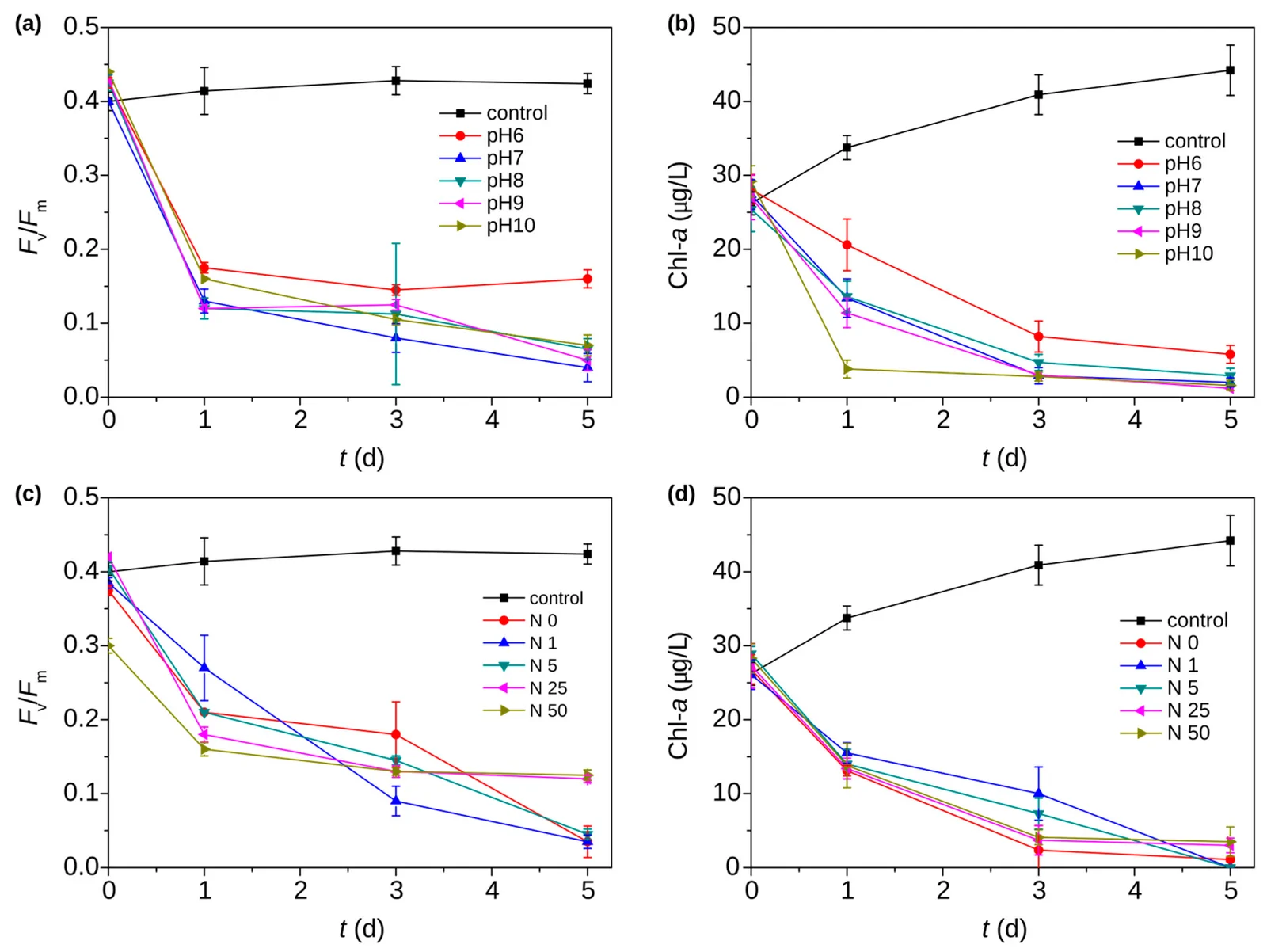
Effects of pH (**a**,**b**) and nitrogen concentration (**c**,**d**) on F_v_/F_m_ and chlorophyll-a in a natural *Microcystis* bloom sample treated with 5 mg L^−1^ of P126-engineered composite. Data are shown as mean ± SD (*n* = 3).

**Figure 6:**
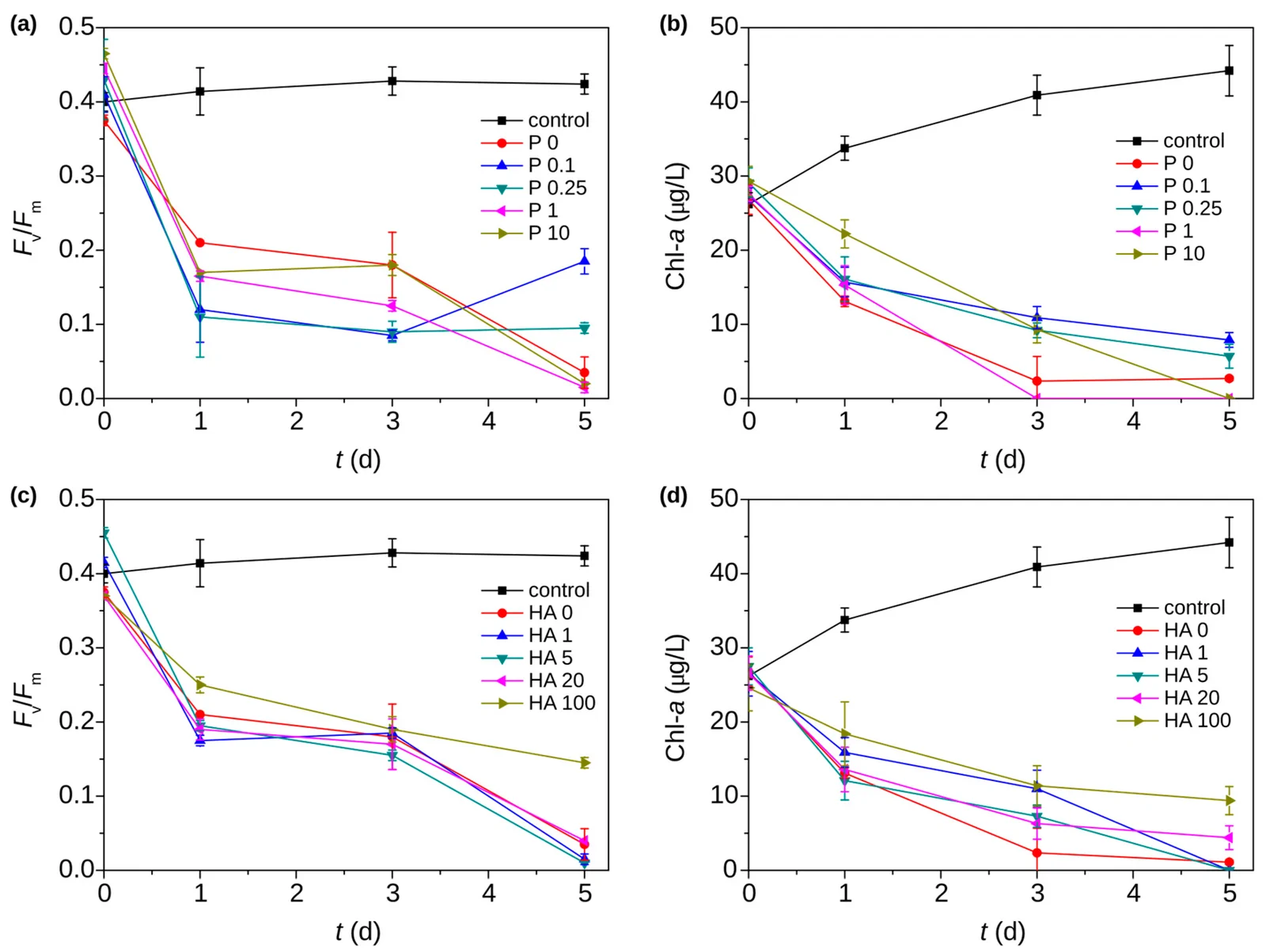
Effects of phosphorus (**a**,**b**) and sodium humate (**c**,**d**) on F_v_/F_m_ and chlorophyll-a in a natural *Microcystis* bloom sample treated with 5 mg L^−1^ of P126-engineered composite. Data are shown as mean ± SD (*n* = 3).

The composite remained active across the tested pH and nutrient ranges (Figure 5 and Figure 6). Suppression was stronger under alkaline conditions, as reflected by lower F_v_/F_m_ and chlorophyll-a values at pH 8–10 than at pH 6 (Figure 5a,b). Variation in nitrogen and phosphorus did not abolish composite-associated suppression, although the trajectories diverged among concentrations at individual time points (Figure 5c,d; Figure 6a,b).

Increasing sodium humate attenuated composite performance (Figure 6c,d). At the highest humate concentration, F_v_/F_m_ and chlorophyll-a remained higher than in the low-humate treatment groups, indicating interference by dissolved organic matter.

### 3.5. Danio Rerio Early-Life-Stage Responses

**Figure 7:**
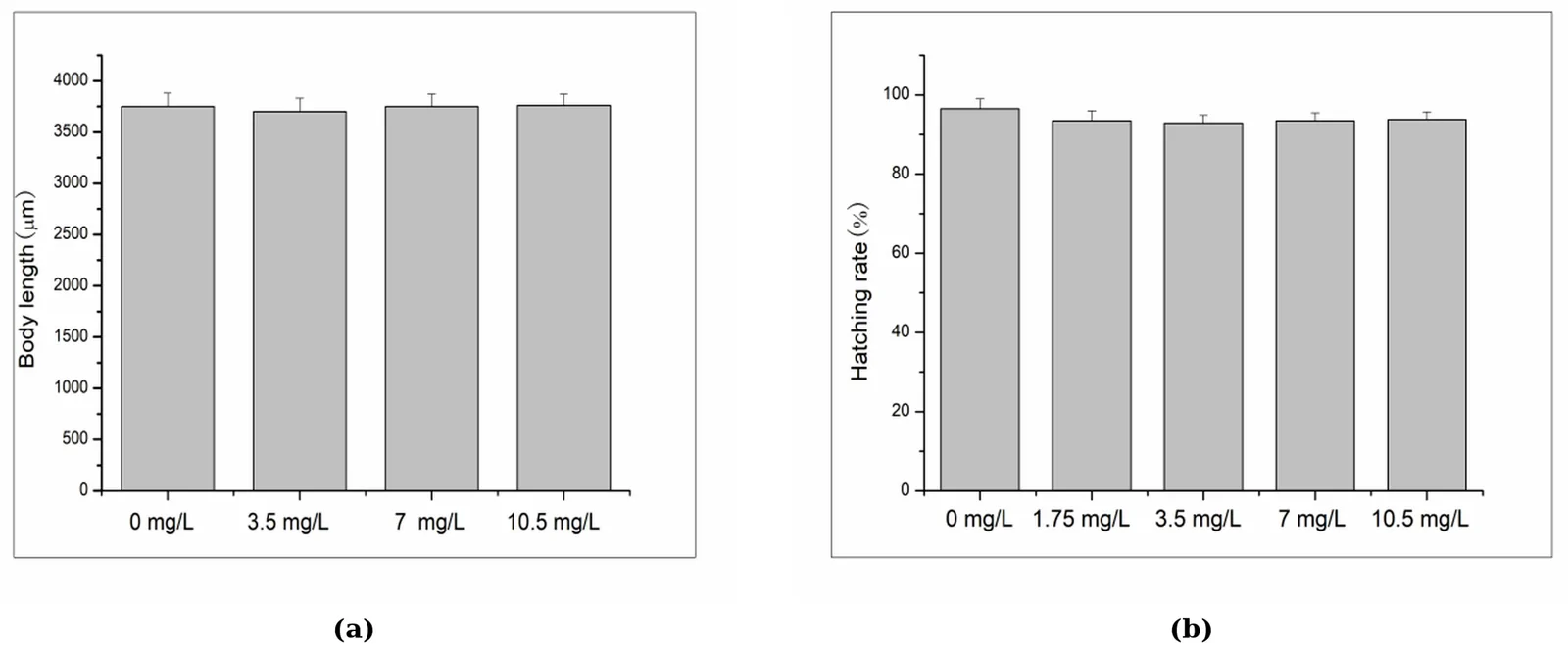
Short-term *Danio rerio* early-life-stage endpoints after exposure to the P126-engineered composite: (**a**) larval body length 96 h post-fertilization at 0, 3.5, 7.0, and 10.5 mg L^−1^; (**b**) hatching success 72 h post-fertilization at 0, 1.75, 3.5, 7.0, and 10.5 mg L^−1^. The exact hours post-fertilization and developmental stage at exposure initiation were not retained in the archived study record; therefore, the exact exposure duration before these endpoints cannot be reconstructed. Data are shown as mean ± SD (30 embryos per replicate; *n* = 3 replicates).

No statistically detectable treatment-related differences were observed in larval body length or hatching success at the tested composite concentrations (exploratory *p >* 0.05; Figure 7). The highest tested concentration was 10.5 mg L^−1^. These findings apply only to the measured short-term pelagic endpoints and exposure conditions; no sediment-dwelling organism was tested.

### 3.6. Field-Scale Ecological Responses

**Figure 8:**
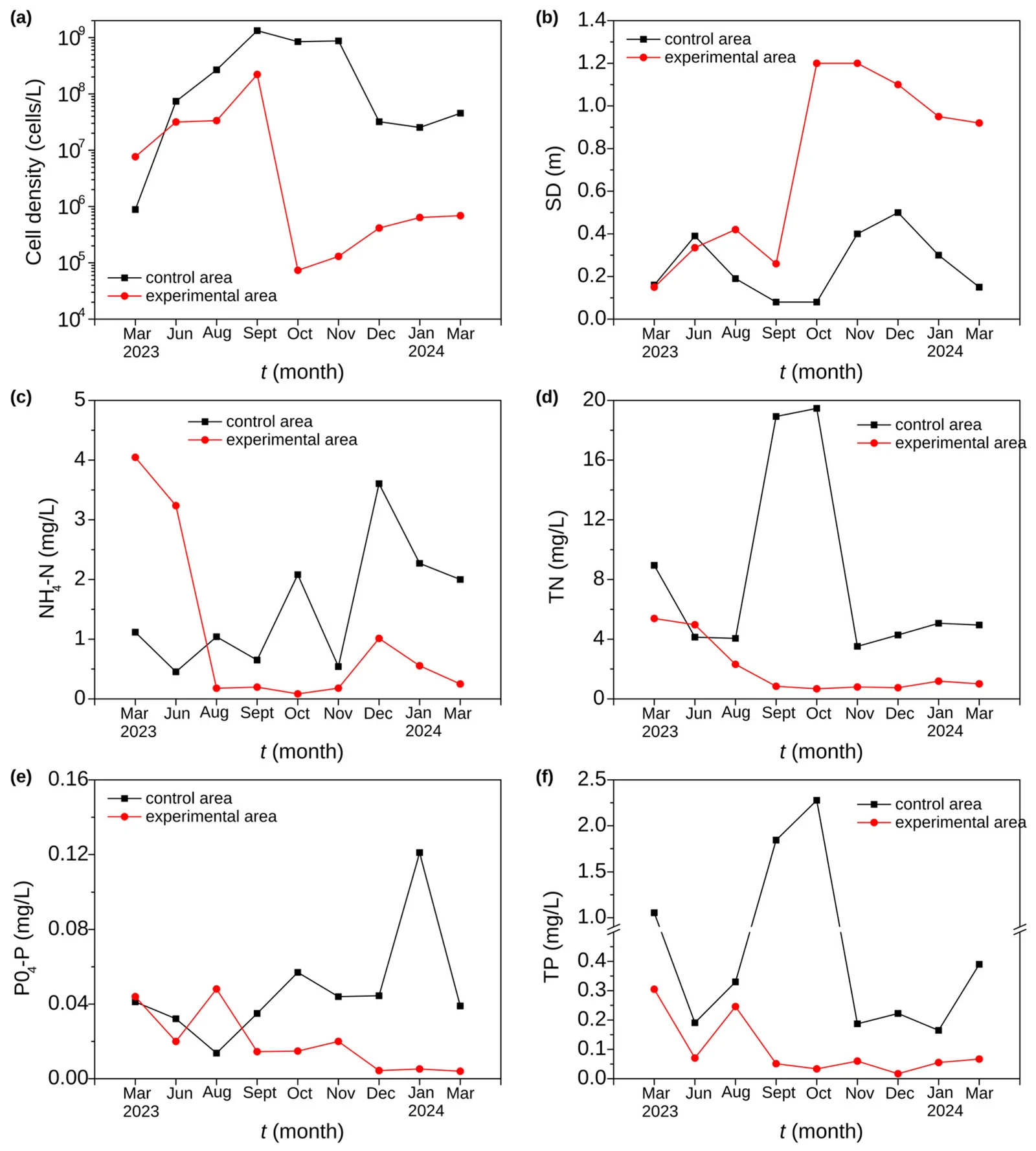
Longitudinal observations in the treated sub-lake and hydrologically separated reference basin from March 2023 to March 2024: (**a**) algal/cyanobacterial cell density, (**b**) Secchi depth, (**c**) NH_4_–N, (**d**) TN, (**e**) PO_4_–P, and (**f**) TP. The field design included one treated and one reference basin; values are descriptive observations, no basin-level inferential test was applied, and repeated dates are not independent basin replicates.

Composite application in September 2023 was followed by an observed reduction in cyanobacterial/algal abundance in the treated basin (Figure 8a). The plotted density decreased from approximately 2 × 10^8^ cells L^−1^ in September to <10^5^ cells L^−1^ in October, a descriptive reduction of more than three orders of magnitude, while the reference basin remained at high abundance. Secchi depth in the treated basin increased from approximately 0.25 m in September to approximately 1.2 m in October and remained at approximately 0.9 m or greater through the end of monitoring (Figure 8b). These values are observations from one treated and one reference basin, not a statistical trend.

**Figure 9:**
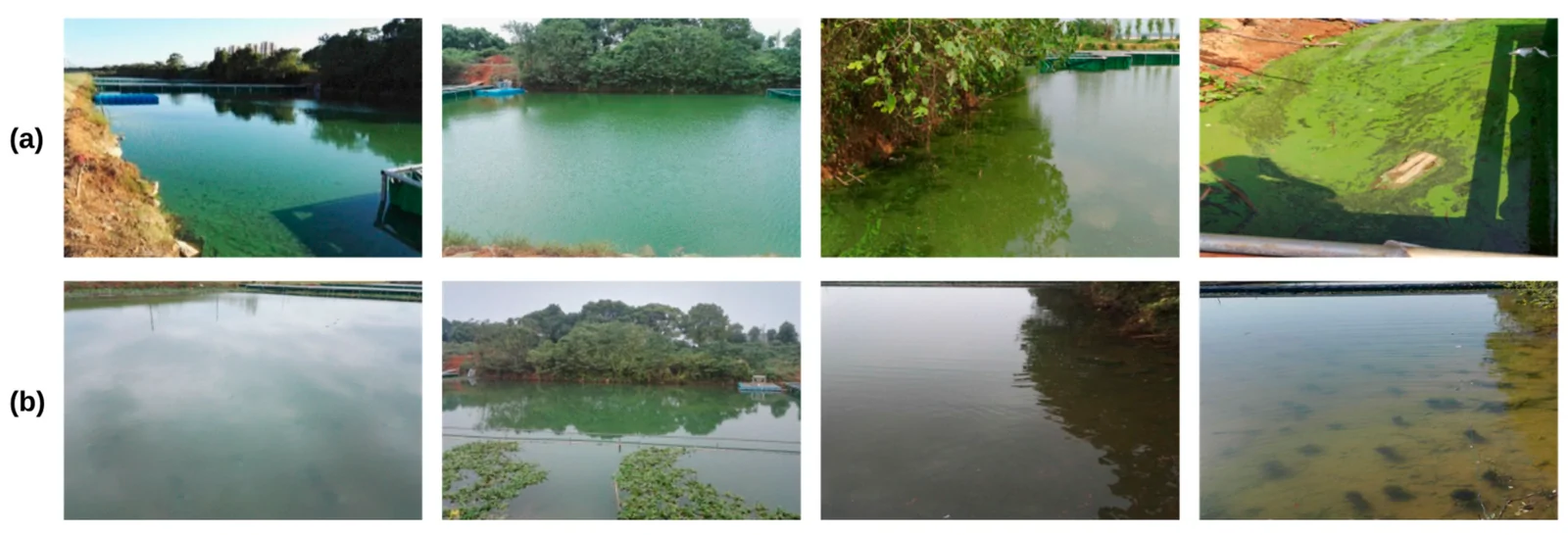
Field photographs documenting conditions (**a**) before composite application and (**b**) after bloom suppression and the subsequent *Vallisneria natans* planting intervention.

From October 2023 to March 2024, the plotted treated-basin ranges were approximately 0.08–1.0 mg L^−1^ for NH_4_–N, 0.6–1.2 mg L^−1^ for TN, 0.003–0.020 mg L^−1^ for PO_4_–P, and 0.015–0.070 mg L^−1^ for TP, compared with approximately 0.55–3.6, 3.5–19.5, 0.04–0.12, and 0.16–2.25 mg L^−1^, respectively, in the reference basin (Figure 8c–f). These are descriptive readings from one basin per condition. *Vallisneria natans* was planted only after the initial clarity improvement. Accordingly, the September-to-October abundance and clarity response preceded the macrophyte-restoration phase, whereas subsequent nutrient and clarity observations reflect the combined influences of composite treatment, hydrological separation, and macrophyte uptake. Their contributions cannot be quantitatively partitioned with the present unreplicated design. Field photographs document the intervention sequence (Figure 9) but do not establish single-factor causality.

## 4. Discussion

### 4.1. Electrostatic Association and Taxon-Dependent Responses

Bloom-forming *Microcystis* commonly produces extracellular polymeric substances (EPS) enriched in deprotonated functional groups, resulting in strongly negative cell-surface potentials [28,29,30,31,32]. P126 modification reversed the charge of the red-soil carrier, creating a large electrostatic contrast with the tested cyanobacteria. The parallel zeta-potential and F_v_/F_m_ results are consistent with preferential association of the positive composite with highly negative cyanobacterial surfaces.

Cationic particles can promote charge neutralization, bridging, and close cell–particle contact [33,34,35]. Such contact may contribute to membrane perturbation or other physiological stress, as reported for polycationic materials [21], but the present study did not directly measure membrane permeability or ultrastructural damage. The mechanism should therefore be interpreted as electrostatically favored association followed by contact- and sedimentation-associated physiological suppression, rather than as direct proof of a specific molecular lesion. Because the reported biological efficacy assays did not include a matched unmodified-soil treatment arm, the incremental contribution of P126 functionalization relative to carrier-associated settling or contact cannot be quantified independently.

Preserving chlorophyte and diatom functions is ecologically relevant because phytoplankton traits and morphology influence grazer interactions and trophic transfer [36,37]; however, the limited responses of four tested strains define selectivity only within this set. Surface chemistry can vary with species, growth phase, medium composition, and natural organic matter. Broader taxonomic testing and mixed-community experiments are needed before phylum-wide selectivity is inferred.

### 4.2. Colonial Suppression and Interpretation of Microcystin Data

Large *Microcystis* colonies contain dense three-dimensional EPS matrices that can impede mass transfer and interact with metal ions [29,31]. This provides a plausible explanation for the weak 24 h response to CuSO_4_ in the ≥500-μm fraction. The settling composite can instead enmesh colonies and create multiple local contact points, which is consistent with its faster MTT-based suppression. Because Cu^2+^ penetration and polymer distribution within colonies were not measured directly, this explanation remains mechanistic interpretation rather than demonstrated transport kinetics. Moreover, the composite and CuSO_4_ were compared at equal nominal mass concentrations rather than on an equimolar or active-component basis; the comparison should therefore be regarded as an operational efficacy benchmark rather than a potency-normalized comparison.

Because the ELISA aliquots were analyzed without prior fractionation, the lower signal after composite treatment may reflect differences in cell disruption, adsorption or complexation within settling flocs, retention of cell-associated toxin, matrix-dependent assay accessibility, or a combination of these processes [38,39,40]. Combined bloom-and-toxin removal or degradation has been reported for other treatment systems [41,42], but the present experiment did not isolate those mechanisms. ELISA is immunoreactive rather than congener-resolved, and the comparison therefore supports only a lower unfractionated-sample microcystin-equivalent signal, not reduced cell lysis, reduced dissolved-toxin release, or lower congener-specific toxicity. Future studies should combine paired whole-sample, supernatant, and pellet measurements with validated LC–MS/MS analysis and water–sediment mass balance.

### 4.3. Environmental Compatibility, Residual Uncertainty, and Application Boundaries

The environmental persistence and biological activity of quaternary ammonium compounds and related cationic polymers are legitimate concerns [25,26]. The two post-preparation water washes were intended to reduce readily soluble P126, and the retained positive zeta potential is consistent with cationic surface functionality after processing. These observations do not establish complete immobilization. No P126-specific analytical method, detection or quantification limit, polymer mass balance, ionic-strength-dependent desorption test, transformation-product measurement, or sediment-residue measurement was available. The 1:4 value is an input formulation ratio before washing, not a measured P126 fraction in the final product. Because post-wash product recovery and polymer mass balance were not determined, the retained P126 fraction—and therefore the actual freely dissolved P126 exposure after field application—cannot be calculated reliably from the nominal composite dose.

The *Danio rerio* assay provides organism-level evidence only for the measured short-term developmental endpoints at concentrations of up to 10.5 mg L^−1^. Waterborne copper can cause developmental and cellular stress in early life stages of zebrafish [43,44], but those dissolved-copper exposures are not directly comparable with the particulate P126-soil suspension used here. Accordingly, the present assay does not establish a chronic no-effect threshold, whole-ecosystem safety, or comparative safety relative to dissolved CuSO_4_. Because no sediment-dwelling organism was tested, the absence of detectable effects in zebrafish embryos cannot be extrapolated to *Hyalella azteca*, *Chironomus* spp., sediment microorganisms, or other benthic receptors.

Because the composite settles, benthic exposure requires particular attention. Priority assessments should include sediment-water survival and growth tests with *Hyalella azteca* and *Chironomus* spp., sediment microbial-community and function endpoints, and chronic water–sediment tests, together with measurement of parent P126 and transformation products. In addition, sedimented cyanobacteria may retain some capacity to survive or re-enter the water column. The observed physiological suppression may reduce this risk, but it does not demonstrate complete elimination of the benthic inoculum.

### 4.4. Operational Robustness and Interpretation of Field Responses

The stronger treatment response at alkaline pH is consistent with increased deprotonation of cyanobacterial EPS and a larger electrostatic contrast with the positive composite [30,31,32]. Variation in nitrogen and phosphorus did not eliminate treatment activity within the tested ranges, whereas humic substances reduced performance, likely through competition for cationic sites and electrostatic shielding [26,40]. Field dosing should therefore be site-specific and should account for dissolved organic matter, conductivity, bloom density, and colony morphology rather than relying on a universal nominal concentration. The 11.25 mg L^−1^ per-application field dose was an operational choice and should not be interpreted as a general environmental safety threshold.

The field observations indicate that composite application was followed by an initial optical window associated with lower plotted algal/cyanobacterial cell density and greater water clarity. *Vallisneria natans* was planted only after this initial improvement. Submerged macrophytes can reinforce clear-water conditions, while successful establishment is strongly constrained by underwater light availability [45,46,47]. Consequently, the September-to-October response can be described as an immediate post-composite observation, whereas the longer-term nutrient and clarity trajectory reflects the combined influences of composite application, hydrological isolation, and active macrophyte restoration. Seasonal forcing and other unmeasured basin-specific differences also cannot be excluded. The present design cannot quantitatively separate these contributions.

The field component also lacked replicated treated basins and did not consistently resolve percentages of chlorophytes and diatoms or responses of zooplankton, benthos, fish, and sediment microorganisms. The time series therefore documents a site-specific operational response rather than a definitive ecosystem-safety experiment. Changes in unmeasured community components may have important food-web consequences [36,37].

### 4.5. Study Limitations and Research Priorities

Limitations affect the interpretation of this study. First, no polymer-specific method was used to quantify freely dissolved, desorbed, or transformed P126, and no validated detection limit or ionic-strength-dependent desorption dataset was available. Second, the reported biological efficacy assays did not include a matched unmodified-soil treatment arm, limiting quantitative separation of carrier-associated effects from those of P126 functionalization. Third, the ELISA did not provide congener-specific microcystin data or dissolved–particulate fractionation. Fourth, short-term zebrafish endpoints did not address chronic or benthic effects, and the exact hours post-fertilization and developmental stage at zebrafish exposure initiation were not retained. Also, the field case study involved one treated and one reference basin, included a subsequent macrophyte-planting intervention, and cannot exclude seasonal or basin-specific confounding. A next-stage evaluation should use replicated enclosures or mesocosms with untreated, raw-soil carrier, and P126-engineered composite treatments; quantify P126 and transformation products in water and sediment across pH and ionic-strength gradients; apply LC–MS/MS to paired whole, dissolved, and particulate microcystin fractions; initiate and document zebrafish exposure at a defined developmental stage; and monitor pelagic and benthic trophic levels over ecologically relevant periods. The composite should be considered a candidate emergency bloom-management tool within an integrated strategy, not a substitute for long-term nutrient-load reduction and ecosystem management.

## 5. Conclusions

This study developed a P126-engineered local-soil composite that combined a positive surface charge with the sedimentability of a natural mineral carrier. Across the tested strains, the composite preferentially suppressed cyanobacteria, showed strong MTT-based activity against large *Microcystis* colonies, and produced a lower maximum unfractionated-sample ELISA microcystin-equivalent signal than CuSO_4_ under the tested conditions. The ELISA result does not demonstrate reduced cell lysis or reduced dissolved-toxin release. No statistically detectable effects were observed in the measured *Danio rerio* early-life-stage endpoints at concentrations of up to 10.5 mg L^−1^, but these endpoints do not establish chronic or benthic safety.

In the field case study, composite application was followed by a rapid decline in plotted algal/cyanobacterial cell density and increased water clarity, after which *Vallisneria natans* was planted as an additional restoration measure. The immediate and longer-term observations should therefore be interpreted separately. These findings support a mechanism-informed approach with field case-study support for selective bloom suppression. Broader application, however, requires direct measurement of residual P126 and desorption, sediment and chronic ecotoxicity, congener-resolved and fractionated toxin analysis, and replicated multi-trophic field validation.

## Data Availability

The original contributions presented in this study are included in the article/Supplementary Material. Further inquiries can be directed to the corresponding authors.

## Supplementary Materials

The following supporting information can be downloaded at https://www.mdpi.com/article/10.3390/microorganisms14092111/s1, Table S1: Characteristics of raw soil; Table S2: Fe, Cu, Mg, and total P contents of raw soil.
