# Supplementary material for: Field-Scale Suppression of Cyanobacterial Blooms by Cationic Polymer-Modified Local Soil: Selective Inactivation Mechanism and Ecological Responses
Source: Microorganisms. 2026 Sep 21;14(9):2111. doi: 10.3390/microorganisms14092111 (PMC13609796; doi:10.3390/microorganisms14092111)
Supplement: Supplementary file 1 [file microorganisms-14-02111-s001.zip › microorganisms-4550934-supplementary.pdf]

Supplementary Materials

Field-scale suppression of cyanobacterial blooms by cationic polymer-modified local soil: Selective inactivation mechanism and ecological responses

Ningyan Peng[1], Yuanyuan Fang[1], Ping Yang[1], Jin Liu[2], Yunlu Jia[2],Zhengwei Dai[3], Xiaojin Zhang[3]*,Renjie Zhao[4], Shouchun Li[4] , Guofei Dai[1]*

[1] Jiangxi Academy of Water Science and Engineering, Key Laboratory of Watershed Soil and Water Conservation, Technology Innovation Center for Ecological Water Engineering in Poyang Lake, Nanchang 330029, China

[2] Institute of Hydrobiology, Chinese Academy of Sciences, Wuhan 430072, China

[3] Faculty of Materials Science and Chemistry, China University of Geosciences, Wuhan 430074, China

[4] College of Life Science, Jiangxi Normal University, Nanchang 330022, China

* Correspondence: zhangxj@cug.edu.cn (X. Zhang); daiguofei@whu.edu.cn (G. Dai).

Table S1. Characteristics of raw soil

pH	Cation-exchange capacity (cmol kg^{-1})	Organic matter (g kg^{-1})	Sand (%)	Clay (%)	Silt (%)	Source
6.21	10.25	4.65	21.87	44.70	29.43	This study

Table S2. Fe, Cu, Mg, and total P contents of raw soil

Element	Content (w/w%)
Fe	4.481
Cu	0.004
Mg	0.421
Total P	0.022
